# *“God is my only health insurance”*: a mixed-methods study on the experiences of persons with disability in accessing sexual and reproductive health services in Ghana

**DOI:** 10.3389/fpubh.2023.1232046

**Published:** 2023-07-20

**Authors:** Abdul-Aziz Seidu, Bunmi S. Malau-Aduli, Kristin McBain-Rigg, Aduli E. O. Malau-Aduli, Theophilus I. Emeto

**Affiliations:** ^1^Public Health and Tropical Medicine, College of Public Health, Medical and Veterinary Sciences, James Cook University, Townsville, QLD, Australia; ^2^Department of Population and Health, University of Cape Coast, Cape Coast, Ghana; ^3^College of Medicine and Dentistry, James Cook University, Townsville, QLD, Australia; ^4^School of Medicine and Public Health, University of Newcastle, Newcastle, NSW, Australia; ^5^School of Environmental and Life Sciences, University of Newcastle, Newcastle, NSW, Australia; ^6^World Health Organization Collaborating Center for Vector-Borne and Neglected Tropical Diseases, James Cook University, Townsville, QLD, Australia

**Keywords:** barriers, disability, enablers, Ghana, sexual and reproductive health

## Abstract

**Background:**

Access to sexual and reproductive health (SRH) services is a fundamental human right, but people with disabilities (PwDs) in low-and middle-income countries often face multiple barriers to utilisation. This study aimed to assess the level of SRH services utilisation and the enabling and inhibiting factors among PwDs in Ghana’s Ashanti region.

**Methods:**

A sequential explanatory mixed-methods study design was employed, involving quantitative (*n* = 402) and qualitative (*n* = 37) data collection from PwDs. Quantitative data were analysed using descriptive and inferential statistics, while qualitative data were analysed using inductive thematic analysis.

**Results:**

The study found that only 33.8% of the PwDs had ever used SRH services. Utilisation was associated with sex, marital status and travel duration to health facility. The qualitative data revealed that factors at the individual, family/community and health facility levels influenced utilisation of SRH services, acting as both enablers and barriers.

**Conclusion:**

PwDs had relatively low utilisation of SRH services in Ghana’s Ashanti region. To increase utilisation, it is recommended to address the stigma and discrimination towards PwDs, provide more training for healthcare providers, improve the accessibility of healthcare facilities, and strengthen the national health insurance scheme. Further research could explore PwDs’ SRH outcomes and strategies to improve these outcomes in Ghana.

## Background

Persons with disabilities (PwDs) are individuals who have long-term physical, mental, intellectual or sensory impairments which in interaction with various barriers may hinder their full and effective participation in society on an equal basis with others (1). Achieving universal health coverage (UHC) is a critical goal of the Sustainable Development Goals (SDGs) agenda (2). At the 2019 United Nations (UN) general assembly meeting, heads of state reiterated their dedication to SDG 3.8 on UHC, which aims to ensure easy access to sexual and reproductive health (SRH) services and information for all (2). However, disability-related issues have not been adequality incorporated in the UHC despite its commitment to leaving no one behind (3–5).

Globally, approximately 1.3 billion individuals live with disabilities, and among them, 190 million have major difficulties carrying out their daily activities (6). The prevalence of disability is higher in low- and middle-income countries (LMICs) than high-income countries. In LMICs, about 400 million people are disabled, and Africa is home to 80 million of these people (6). In Ghana, the Ghana Statistical Service (GSS) reported that 8% of the population is living with disabilities (7).

SRH is a fundamental aspect of overall health and well-being (8). The World Health Organization (WHO) recognises the importance of SRH for all and has emphasized the need for equal access to SRH services. However PwDs face significant barriers to accessing SRH services and information, which violates their right to health and equality. Therefore, access to SRH services, needs to be addressed to ensure that PwDs have an equal opportunity for sexual health (9). This right is recognized by the United Nations Convention on the Rights of Persons with Disabilities, which states that “PwDs should have access to the same range, quality and standard of free or affordable health care and programs as other persons, including SRH” (1).

Despite this, disability-related issues have not been adequately incorporated into SRH policies and practices (10–12). This neglect can be attributed, in part, to the belief that PwDs are less likely to engage in sexual activity, marry, or have children (13–15). However, research indicates that the SRH needs and desires of PwDs are similar to those without disabilities (6, 14, 16).

PwDs often encounter various barriers hindering their access to SRH services such as antenatal care, contraception, HIV testing, and SRH information. These barriers include economic challenges, physical inaccessibility, disability-insensitive healthcare services, and negative attitudes of health providers (HPs) and community members (17–20). Consequently, PwDs are less likely to use SRH services and are at higher risk of experiencing adverse SRH outcomes (21). Previous studies have explored challenges faced by young PwDs (22, 23) and specific populations such as women with disabilities (20), but a few have examined the enablers and barriers to the utilisation of SRH services among PwDs in LMICs (24). This represents a lost opportunity to unearth the difficulties PwDs face in accessing SRH services. This gap hampers the attainment of SDG 3 and the UHC agenda. Understanding these access issues from the perspectives of those who are most impacted by them is essential. This paper seeks to address this gap in knowledge and contribute to improving the accessibility of SRH services for PwDs. Therefore, this study employs a mixed methods approach to examine the barriers and enablers to SRH services and interventions utilisation among PwDs in Ghana’s Ashanti region. Specifically, the study aims to answer two research questions: (1) What is the level of SRH service use among PwDs? and (2) What are the enablers and barriers to the utilisation of SRH services and interventions among PwDs in Ghana’s Ashanti Region?

### Theoretical framework

**Figure 1:**
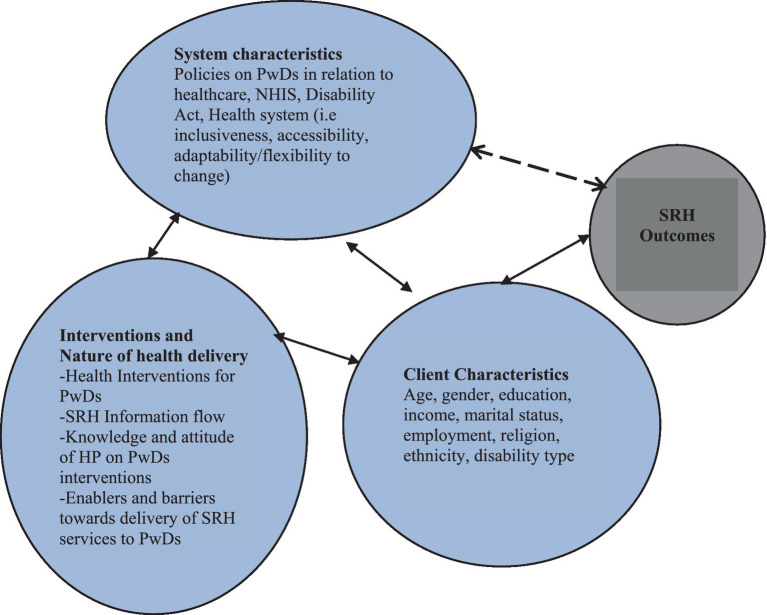
Health outcomes model. Source: Adopted from Mitchell et al. (25) and Radwin (26).

This study is part of a larger mixed methods project entitled “*Impact of health policies and interventions on the sexual and reproductive health outcomes among persons with disabilities in Ghana*.” The theoretical framework guiding this study is the health outcomes model (Figure 1) proposed by Mitchell et al. (25) and Radwin (26). The model posits that client characteristics, systems characteristics and the interventions or nature of health delivery interact to produce positive health outcomes. The framework’s specific components are discussed in the subsequent sections.

### Client characteristics

Client characteristics encompass individual factors, including demographic attributes, that can significantly impact various aspects of a person’s life. In the context of this study, client characteristics pertain specifically to socio-demographic characteristics of PwDs, such as age, gender, type of disability, place of residence, marital status, educational level, religion, and ethnicity. These characteristics influence PwDs’ utilization of SRH services and may also affect their SRH outcomes.

### System characteristics

In this study, the system characteristics refer to how hospitals or HPs networks are perceived as organizational entities, and how these attributes combine to influence health and behavior. The system characteristics examined in this study include the nature of the healthcare system, its inclusivity, accessibility, adaptability/flexibility to change, health administration, and the policies related to PwDs and their SRH. Additionally, the nature of health delivery, encompasses specific services provided by HPs for PwDs, the flow and access of SRH information, the knowledge, and attitudes of HPs towards PwDs, and how these attributes function as enablers or barriers to the use of SRH services among PwDs are also explored.

### Interventions

The interventions are another component of the health outcomes model that this study examines. These are the direct and indirect activities that aim to improve the SRH of PwDs. Some examples of the interventions in this study are the national health insurance scheme (NHIS), free maternal healthcare policy, the Disability Act 715 and the disability common fund (12, 27).

#### Outcomes

From the framework, the outcomes in this study comprise both positive and adverse SRH outcomes that a PwDs may experience including STIs, unintended pregnancy, self-rated SRH, sexual safety, sexual autonomy, sexual satisfaction, and experience of sexual violence (28).

This paper focuses on only three aspects of the framework: system characteristics, interventions or nature of health delivery, and individual or client characteristics, which influence the use of SRH services. The framework posits that the use of health services is determined by the interaction between the characteristics of the population and the healthcare system (29). The framework has been validated (26) and applied in various settings and populations (30), making it suitable for this study. It also allows for the analysis of health outcomes in relation to system, intervention, and individual characteristics (30) which are the key issues addressed in the main study (Figure 1). The system characteristics, interventions, and client characteristics sections have been employed to assess HPs attitudes toward PwDs and the enablers and barriers they encounter in delivering SRH services to PwDs (31).

## Methods

### Ethics approval

This study followed the ethical guidelines and approval from three institutional review committees: the Ghana Health Service (GHS) Ethics Review Committee (GHS-ERC: 005–0621), the Komfo Anokye Teaching Hospital (KATH; KATH-IRB/RR/101/21), and the James Cook University (JCU) Human Ethics Committee (H8531). Additionally, the Regional Health Directorate in Kumasi and the Offinso North District Health Directorate in Akumadan also endorsed the ethics approval forms. The leaders of two disability groups in Kumasi Metropolis and Offinso North District also consented to the study. Furthermore, participants’ anonymity and confidentiality were ensured. Specifically, before the administration of the study instrument, a document stating the study objectives, anonymity, confidentiality and merits of the study were explained to respondents. The respondents were made aware that the information provided is purely for academic work and that their identities will not be revealed to the general public. Respondents were also informed about their right to withdraw from the study at any given time if they so desire. Written and verbal informed consent were sought from all the respondents.

### Study design, population, and setting

**Figure 2:**
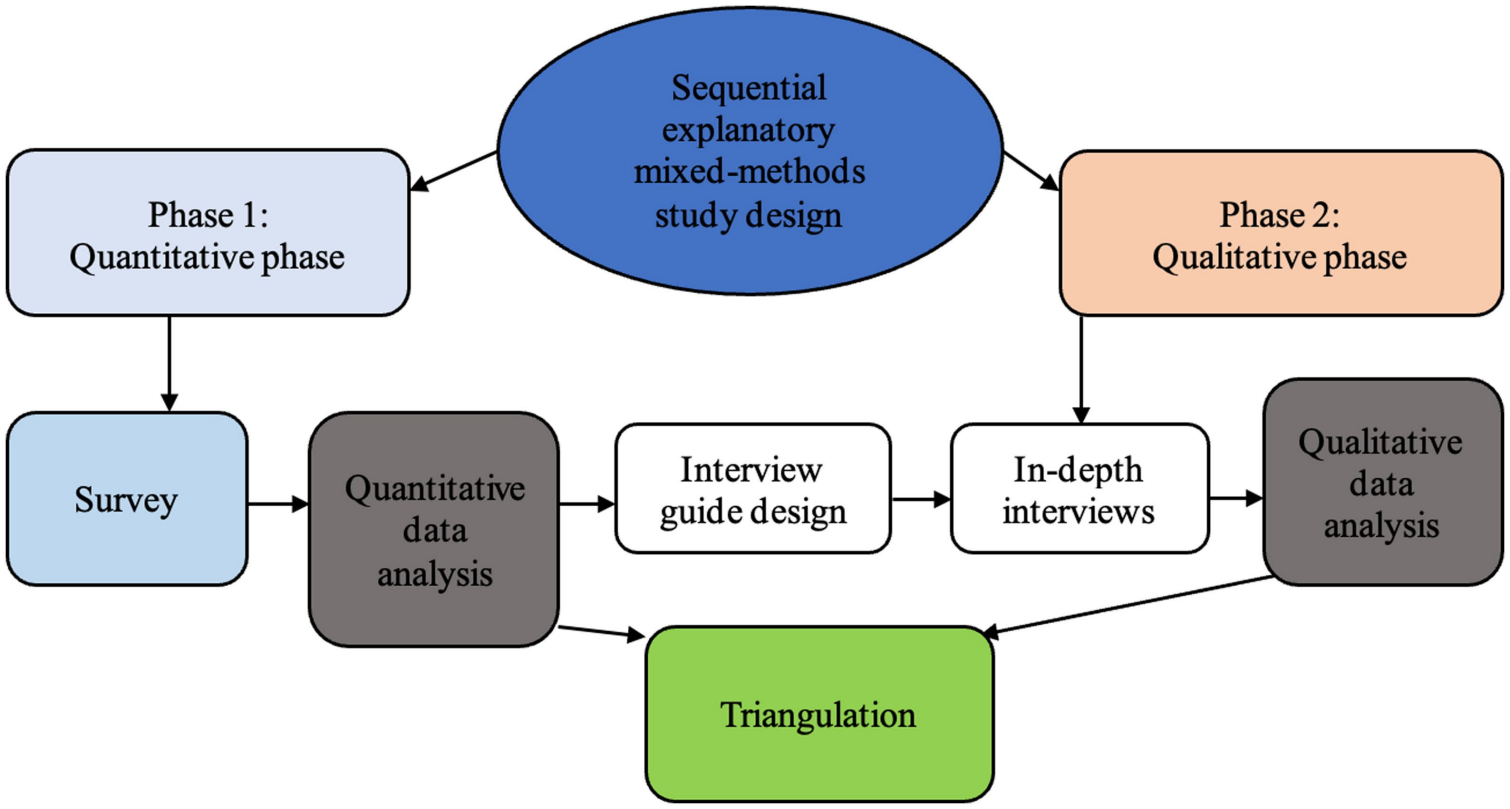
Sequential explanatory study design (32).

The study adopted a sequential explanatory mixed-methods (32) approach (Figure 2) underpinned by the pragmatic paradigm to collect data from PwDs in the Ashanti Region (Kumasi Metropolis and Offinso North District) of Ghana. It is situated in the central belt between longitudes 0.15° W and 2.25° W and latitudes 5.5° N and 7.46° N. It shares borders to the north, south, east, and west with the Bono East, Central, Eastern, and Western Regions, respectively. It occupies around 24,389 square kilometers, or 10.2% of Ghana’s land. The Ashanti region has an urban population of about 61.6%. The study setting was selected based on the 2021 Population and Housing Census report, which indicated that the Ashanti Region had the highest percentage (17.3%) of PwDs in Ghana. The most common categories of disabilities in this region were visual/seeing (4%) and physical impairment/walking (3.6%), with more PwDs living in urban (9.5%) than rural areas (6.5%) (7). Detailed description of the study area is published in a previous study (31). The inclusion criteria were: (a) participants must be 18 years and above b) participants with physical disability or vision impairments. The exclusion criteria were: a)participants less than 18 years, (b)Participants with multiple disabilities, (c)participants with other forms of disability.

### Data collection

Four trained Research Assistants (RAs) with expertise in survey data collection and disability issues were involved in the data collection. They received a five-day training using a designed training manual. The quantitative data collection took place from January 10 to April 24, 2022, while the qualitative data collection took place from May 5 to July 11, 2022.

### Phase one: quantitative phase

#### Sample size and sampling

The study used the formula by Lwanga et al. (33) to determine the sample size of 402 PwDs. It is given as 𝑛 = z^2^ 𝑝𝑞/𝑑^2^ where *n* = sample size, *p* = proportion of PwDs use of SRH services, d = level of uncertainty (5%/0.05), z^2^ = 95% level of confidence and 𝑞 = 1−𝑝. The researchers compiled a list of PwDs (Visual and Physical disability) by contacting their group leaders. A systematic sampling technique–a quantitative sampling approach where the initial unit is randomly selected and subsequent selection is based on a fixed sampling interval from the random start point (34) was applied to select the respondents from the list, who were then contacted during their weekly meetings. Specifically, the list of PwDs served as the sampling frame. The sampling interval was established using the sampling frame by dividing the total number of PwDs by the minimum required sample size for the study. Commencing with the initial 10 names within the sampling frame, a respondent was randomly chosen, serving as the starting point for the sampling process. Subsequently, respondents were selected at regular intervals determined by the sampling interval until the desired sample size was achieved.

#### Data collection instrument and procedure

The primary data were collected using a questionnaire (Appendix 1) based on previous validated instruments (35) and literature review (19, 20, 24). The questionnaire consisted of several sections; however, only two primary sections were utilized to meet the specific objectives of this study. These sections included the client characteristics section, which examined various demographic and personal attributes of the participants, and the uptake of SRH services and interventions section, which focused on the participants’ utilization of SRH services. The questionnaire was administered in Twi, the predominant language in the study area. Four experienced RAs from the Department of Health Promotion, Education and Disability Studies, Kwame Nkrumah University of Science and Technology, Kumasi, Ghana, and the Department of Population and Health, University of Cape Coast, Ghana were recruited and trained 3 days with a manual on how to ask questions, seek consent, and adhere to ethical principles. The questionnaire was pretested among 30 PwDs in Nkawie and Nkenkaasu before the data collection, which took an average of 18 min per questionnaire. After the pre-test, there was no modification to the questionnaire.

#### Statistical analyses

The study used descriptive statistics, such as frequencies and percentages, to describe the socio-demographic characteristics of the respondents. It also employed binary logistic regression analysis to assess the factors associated with the use of SRH services among PwDs. The dependent variable was the use of SRH services (yes or no), and the independent variables were the socio-demographic characteristics and disability-related factors. The study set the level of statistical significance at *p* < 0.05. All the statistical analyses were performed using Stata version 14 (StataCorp SE, College Station, TX, USA) (36).

### Phase two: qualitative phase

To explore the issues identified in the first phase of the study, an interview guide (Appendix 2) was designed to elicit in-depth qualitative data from the participants to gain deeper understanding of their SRH service utilisation. A purposive sampling technique of consenting participants from the first phase of the project with maximum variation was used to select 37 PwDs from the two study sites, ensuring a balance of gender/sex and type of disability. The lead author (A-AS) and a female RA conducted face-to-face interviews with the participants at agreed private convenient places such as participants homes and weekly meeting venues. The RA received a two-day training on the qualitative research objectives and interviewing skills using a manual developed for this purpose. Each training session lasted for 120 min. The interview guide was piloted among four PwDs in Nkawie (2) and Nkenkaasu (2) to check for clarity and comprehension of the questions. The pilot revealed that there was no equivalent term for SRH in Twi (the local language), so the concept had to be explained to the participants. The interviews lasted for an average of 57 min. Verbal and written consent were obtained from the participants before the interviews. All interviews were audio-recorded and supplemented by field notes that captured non-verbal cues. Data saturation is achieved in qualitative data collection when no additional issues or insights are identified (37). This occurs when participants repeatedly mention the same themes and concepts. In the present study, data saturation was reached after the 35^th^ participant. However, two additional interviews were conducted with participants who had previously expressed interest in the study to prevent the unintentional elimination of information. The data collection was therefore stopped after the 37^th^ participant.

#### Trustworthiness

Trustworthiness was upheld by following the key strategies proposed by Lincoln and Guba (38). These include credibility, dependability, confirmability, and transferability (39). Credibility was upheld by ensuring that interviewers (RAs) were well trained with the required skills and knowledge to conduct the interviews. Interview guide was pretested before actual data collection; interviews were conducted at convenient places proposed and agreed by both the interviewers and participants; as well as regular debriefing sessions to discuss issues after each day’s work (39). The researchers used the local language, Twi for participants to easily express themselves. The researchers adopted various strategies to ensure honesty in information the participants gave. This was ensured by asking the participants to be frank in their responses and rephrasing questions differently to elicit same or similar responses. Again, there was the use of probes and iterative questioning and the development of early familiarity with the participants through the first phase of the project (40). Dependability was ensured by preparing a detailed draft of the study protocol, keeping detailed track records of all the data collection processes and ensuring that there was coding accuracy, verified by all the research team members (41). Confirmability was obtained through, checking by supervisors, co-coding and the confirmation of identified themes by all research team members. In addition, several triangulation techniques were employed. For example, both qualitative and quantitative data were collected from different participants (different groups of PwDs from two districts). To ensure transferability, this paper provides a comprehensive description of the study methodology, allowing for potential replication and follow-up by other researchers. The research design, including the selection of the study setting, as well as the utilization of the purposive sampling technique to identify participants meeting the predefined inclusion criteria, have been elaborated upon in detail. These methodological aspects serve to enhance the transferability and replicability of the study, facilitating the application of its findings to other contexts (39).

#### Qualitative data analysis

The data analysis was conducted using NVivo version 12 and following an inductive thematic analysis approach (42). The lead author (A-AS) and the RA transcribed all the audio recordings verbatim and checked them for accuracy against the original interviews. The transcripts were then read and coded by A-AS, with guidance from KM-R, and TE, who met regularly to discuss the analytical framework and direction. The codes were then collated into potential themes or key topics by A-AS, KM-R, and TE, who also reviewed the themes for coherence and relevance. The final themes and illustrative quotes were confirmed by the team. The themes were presented with their corresponding demographic information of the participants, such as sex, age, type of disability and district, using verbatim quotes. The quality of the qualitative report was assessed using the Consolidated criteria for reporting qualitative research (COREQ) checklist (Appendix 3) (43).

## Results

### Phase one: quantitative results

#### Socio-demographic characteristics of respondents

The socio-demographic characteristics of the 402 respondents and their level of SRH utilisation are presented in Table 1. The respondents were predominantly visually impaired (57.7%), male (51.5%), urban dwellers (80%), Akans (82%), Christians (88.6%), and NHIS subscribers (96.8%). About 30% of them were aged 60 and above, 34% had senior high school/tertiary level of education, and 50% were employed. The majority (44%) were married.

**Table 1:** Socio-demographic characteristics of respondents and sexual and reproductive health services utilisation (*n* = 402).

Variable	Frequency	Percentage	Sexual and reproductive health services usage	
			No (66.2%)	Yes (33.8%)
Disability type			*χ*^2^ = 71.1, *p* = 0.008	
Physically disabled	170	42.3	58.82	41.18
Visually impaired	232	57.7	71.55	28.45
Age (Years)			*χ*^2^ = 6.36, *p* = 0.174	
18–29	46	11.4	80.43	19.57
30–39	79	19.7	63.29	36.71
40–49	73	18.2	65.75	34.25
50–59	89	22.1	59.55	40.45
60+	115	28.6	67.83	32.17
Sex			*χ*^2^ = 23.5, *p* < 0.001	
Female	195	48.5	54.36	45.64
Male	207	51.5	77.29	22.71
Residence			*χ*^2^ = 2.77, *p* = 0.096	
Kumasi Metro	323	80.4	68.11	31.89
Offinso North	79	19.7	58.23	41.77
Level of education			*χ*^2^ = 5.3, *p* = 0.15	
No formal education	68	16.9	69.12	30.88
Primary	66	16.4	60.61	39.39
JHS	130	32.3	60.77	39.23
SHS/Tertiary	138	34.3	72.46	27.54
Religious affiliation			*χ*^2^ = 2.28, 0.131	
Christian	356	88.6	64.89	35.11
Non-Christian	46	11.4	76.09	23.91
Marital status			*χ*^2^ = 26.6, *p* < 0.001	
Never married	97	24.1	86.60	13.40
Married	178	44.3	63.48	36.52
Separated/Widowed/Divorced	127	31.6	54.33	45.67
Ethnicity			*χ*^2^ = 1.44, *p* = 0.231	
Akan	330	82.1	64.85	35.15
Non-Akan	72	17.9	72.22	27.78
Employment			*χ*^2^ = 1.26, *p* = 0.262	
Not working	199	49.5	68.84	31.16
Working	203	50.5	63.55	36.45
NHIS Subscription			*χ*^2^ = 0.13,*p* = 0.720	
No	13	3.2	61.54	38.46
Yes	389	96.8	66.32	33.68
Duration to the nearest health facility(Minutes)			*χ*^2^ = 4.81, *p* = 0.090	
Less than 30 min	133	33.1	62.41	37.59
30–59 min	192	47.8	64.58	35.42
60 and above minutes	77	19.2	76.62	23.38

NHIS, National Health Insurance Scheme; SHS, Senior High School; JHS, Junior High School; *χ*^2^ = Chi-square; *p*, *p*-value.

Table 1 also reveals that only 33% of the respondents had ever used SRH services, and this varied significantly across some of their demographic characteristics. The physically disabled (41%) were more likely to use SRH services than the visually impaired (28%). Similarly, females (45.6%), those aged 50–59 (40.5%), those in the Offinso North District (41.7%), those with primary or junior high school level of education (39%), and those who spend less than 30 min to get to a health facility (37.6%) had higher proportion of SRH utilization than their counterparts. The chi-square analysis shows that disability type (*χ*^2^ = 71.1, *p* = 0.008), sex of respondent (*χ*^2^ = 23.5, *p* < 0.001), and marital status (*χ*^2^ = 26.6, *p* < 0.001) were significantly associated with SRH utilization.

### Factors associated with sexual and reproductive health services usage among persons with disabilities

The association between background characteristics and SRH use among PwDs is presented in Table 2. The results indicate that gender, marital status, and travel time to a health facility were significantly associated with SRH use. Male PwDs had lower odds of using SRH services than female PwDs (aOR = 0.29; 95%CI = 0.16–0.52). PwDs who were married (aOR = 5.53; 95%CI = 2.18–14.06), separated or divorced (aOR = 6.15; 95%CI = 2.34–16.18) had higher odds of using SRH services than those who were never married. Furthermore, PwDs who travelled more than 60 min to a health facility had lower odds of using SRH services than those who travelled less than 30 min (aOR = 0.38; 95% CI = 0.17–0.83).

**Table 2:** Factors associated with sexual and reproductive health services utilisation among persons with disabilities.

Variable	aOR	95%CI		*p*-value
Disability type				
Physically disabled	Ref			
Visually impaired	0.60	0.35	1.04	0.068
Age (Years)				
18–29	Ref			
30–39	1.24	0.45	3.42	0.674
40–49	1.03	0.35	3.03	0.958
50–59	1.21	0.39	3.76	0.743
60+	0.99	0.31	3.18	0.982
Female	Ref			
Male	0.29	0.16	0.52	<0.001
Place of residence				
Kumasi Metropolis	Ref			
Offinso North	1.11	0.56	2.19	0.767
Level of education				
No formal education	Ref			
Primary	1.47	0.68	3.20	0.332
Junior High School	1.93	0.91	4.11	0.085
SHS/Tertiary	1.58	0.72	3.47	0.252
Religion				
Christianity	Ref			
Non-Christian	0.97	0.41	2.31	0.951
Marital status				
Never married	Ref			
Married	5.53	2.18	14.06	<0.001
Separated/Widowed/Divorced	6.15	2.34	16.18	<0.001
Ethnicity				
Akan	Ref			
Non-Akan	0.70	0.37	1.33	0.281
Working status				
Not working	Ref			
Working	1.13	0.67	1.91	0.641
NHIS				
No	Ref			
Yes	0.84	0.26	2.72	0.765
Duration to health facility(Minutes)				
Less than 30 min	Ref			
30–59	0.72	0.39	1.29	0.269
60+	0.38	0.17	0.83	0.016

aOR = adjusted odds ratio; Ref = reference category; CI = confidence interval; SHS = Senior High School; NHIS = National Health Insurance Scheme; SRH, sexual and reproductive health.

**Figure 3:**
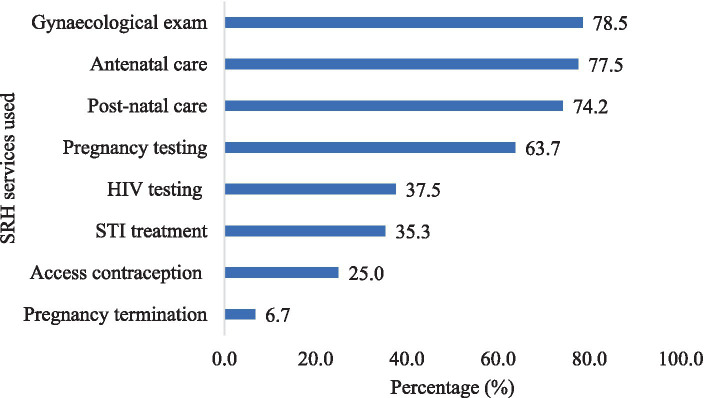
Sexual and reproductive health services used by respondents.

Figure 3 shows the SRH services used by the respondents. The most used service was gynaecological examination, reported by 78.5% of the respondents. Antenatal care and postnatal care were also frequently used, by 77.5% and 74.2% of the respondents, respectively. Only 6.7% of the respondents reported using pregnancy termination services.

**Figure 4:**
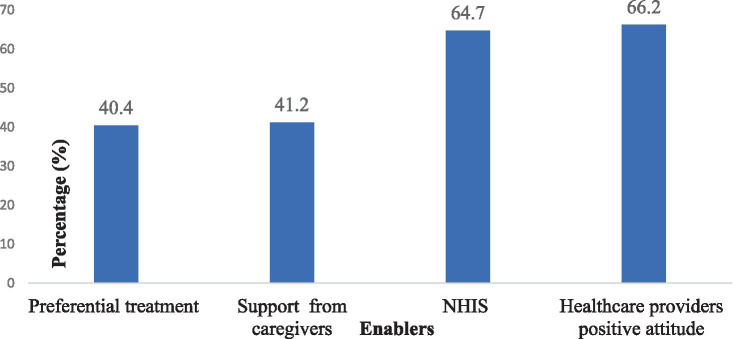
Enablers to sexual and reproductive health services utilisation.

Figure 4 illustrates the factors that facilitated the use of SRH services by the respondents. The most frequently reported enabler was the positive attitude of HPs (66.2%). Another important factor was NHIS subscription (64.7%). Additionally, 41.2% of the respondents indicated that they received support from their care givers, and 40.4% of respondents stated that they benefited from preferential treatment at the health facilities.

#### Barriers to sexual and reproductive health services utilisation

**Figure 5:**
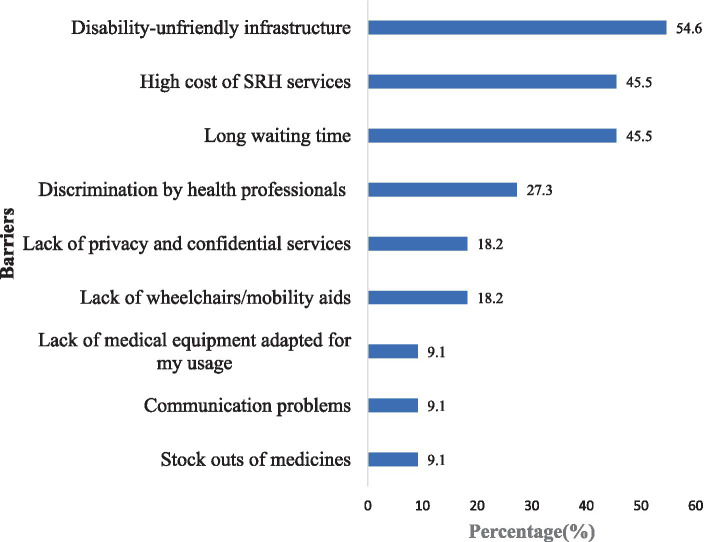
Barriers to sexual and reproductive health services utilisation.

The main barriers to accessing SRH services by PwDs are shown in Figure 5. The results reveal that the lack of disability-friendly infrastructure was the most common barrier, reported by 54.5% of the respondents. Another major barrier was the long waiting time and the high cost of SRH services, which were both cited by 45.5% of the respondents. Moreover, 27.3% of the respondents experienced discrimination by HPs when seeking SRH services.

### Phase two: qualitative results

#### Qualitative results

**Figure 6:**
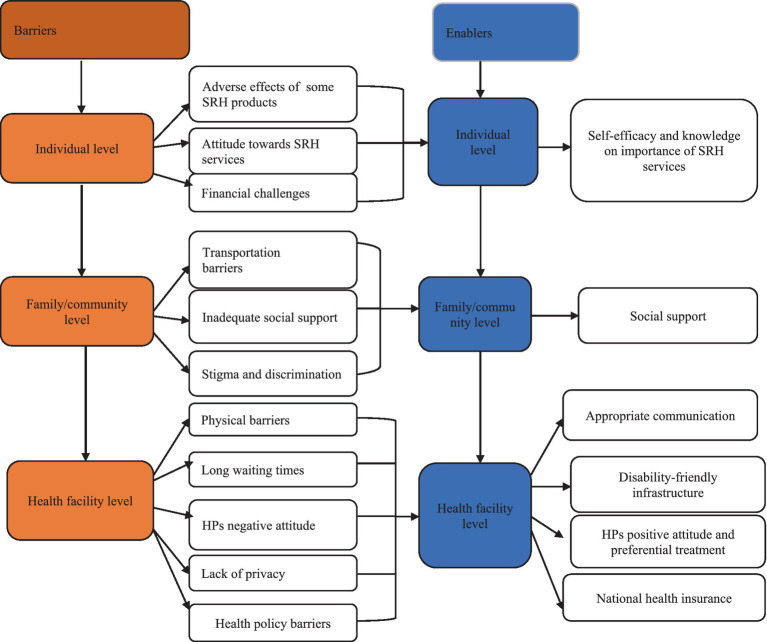
Themes and sub-themes from data.

The qualitative phase of the study involved 37 participants (22 males and 15 females) with ages ranging from 21 to 60 years. Most of the participants were from the Kumasi Metropolis (*n* = 24), belonged to the Akan ethnic group (*n* = 31), identified as Christians (*n* = 34), and were married (*n* = 19). The remaining participants were from Offinso North (*n* = 13) and other ethnic groups (*n* = 6). The analysis of the participants’ experiences of accessing SRH services yielded six overarching themes. Three themes reflected the barriers that hindered their use of SRH services: individual level barriers, family/community level barriers, and facility level barriers. Three themes indicated the factors that enabled their use of SRH services: individual level factors, family/community level factors, and facility level factors. These themes are depicted in Figure 6.

#### Barriers to sexual and reproductive health services usage among persons with disabilities

##### Individual level barriers influencing sexual and reproductive health services usage

In terms of the individual level barriers PwDs indicated that their main barriers to SRH services usage were: a) their experiences of adverse reactions after using some of the SRH products, b) misconceptions and negative attitude towards SRH services and products and c) financial constraints limiting their capabilities to seek SRH services.

###### Adverse effects of some SRH products

A number of the participants shared their personal experiences or how their partners experienced adverse effects after using some family planning commodities.

*“… I tried the injectable but it was affecting me. I was having severe bleeding so, I decided to stop using it. Since then, I have not done any other family planning. Apart from the injectable, there are the oral contraceptive pills which I have used before. But that caused high blood pressure, and so I stopped using that one too. To prevent any further complications, I decided not to use any family planning commodity again.” (Physically Disabled, Offinso, female, 46 years)*

*“…And so, we discussed the issue and decided to go for the five years’ family planning. But when she did it, she started experiencing some weird complications. She was always complaining. So, I took her to the hospital for them to take it out. It was not an easy thing for us. Since that experience, I had that mentality that family planning has adverse health effects on the body and advised her no to even try it again.” (Physically Disabled, Kumasi, Male, 44 years)*

###### Misconceptions and negative attitude towards SRH services

PwDs’ misconceptions and negative attitudes toward SRH services was another individual level barrier influencing the use of SRH services. While this was influenced by some cultural and religious doctrines, other participants mentioned that it was their personal decision not to use SRH services particularly, contraceptives.

*“No, I have never used a condom before because of my religious belief. When you read the Bible, it shows that if you use a condom, it means you are on the same scale as someone who has done an abortion. The sperms are what will create the human and so if you do that, then you have killed your child(ren), which is a great sin”. (Physically Disabled, Offinso, Male, 35 years)*

*“…My religious belief is not a reason for not using some SRH services such as family planning methods. It is my personal preference. I am not enthused about family planning that is why I do not patronize it. Moreover, I have heard about some adverse health effects like bloating and fatigue that are associated with the use of these family planning methods. Some people who use it say that the family planning makes them sick”. (Visually Impaired, Offinso, Female, 54 years)*

###### Financial challenges

A number of the participants also shared how their economic situation prevented them from accessing SRH services. The majority of the participants were unemployed due to their disability status. Those who were employed also earn insufficient amounts of money and hence find it challenging to bear the cost associated with SRH services. Due to this some of the participants reported that they resort to self-medications.

*“With regard to finances, it’s hard for us (PwDs). Me, for instance, I don’t work–unemployed. I was supposed to visit the clinic yesterday but I couldn’t because I didn’t have money. If things were a bit flexible and less costly, I would have visited and had a check-up. So if I’m here and excuse me to say, I pass on, should it be because of money? So, doctors should help us a bit because hospital bills are too high”. (Visually Impaired, Kumasi, Female, 60 years)*

*“Sometimes I feel ill but I cannot go to the hospital because I don’t have enough money on me. Although I believe that if I go to the hospital, I will be fine but healthcare is not free for PwDs so I will stay in the house and rather find some drugs at the pharmacy shop to treat myself”. (Physically Disabled, Kumasi, Female, 27 years)*

###### Family/community level barriers

Another major theme the participants discussed affecting their use of SRH services was family and community level barriers. These barriers comprised challenges with transportation, inadequate social support, and stigma and discrimination from family and community members regarding the SRH rights and capabilities of PwDs.

###### Transportation challenges affecting PwDs use of sexual and reproductive health service

The first community level barrier PwDs shared affecting their access to SRH services was the long distance to health facilities. Participants expressed how certain health facilities were located far away from them and recounted how this hindered their ability to access SRH services.

*“The distance to the healthcare facility is my problem. For example, I had to board a car from where I was to the hospital. Also, at the time that I was pregnant, I was in school. So, you can just imagine. Being a PwD and becoming pregnant while in school; it was a big challenge. People will be like, you are in school as a PwD and you have gotten pregnant. Am I the one to be carrying you to the hospital? So, I decided that I will be going alone sometimes. It was not an easy experience at all but I went through it successfully”. (Visually Impaired, Offinso, Female, 38 years)*

*“Because of my condition, mobility to the place has been a challenge for me... Sometimes, getting a means of transport is a big challenge. So in sum, I will say that mobility is the main challenge for PwDs in this community. It is difficult for me to move around and get means of transportation to go to healthcare facility to access SRH services”. (Visually Impaired, Kumasi, Female, 33 years)*

###### Inadequate social support to persons with disabilities

Another major barrier that emerged from the data at the family/community level was inadequate support from family members. Some of the participants shared how they missed hospital appointments or discontinued SRH service utilization due to unavailable support to access those services.

*“The person who impregnated me did not care at all about the pregnancy. Because of that, I was unable to go for Antenatal care (ANC) services but I eventually delivered at the hospital”. (Physically Disabled, Offinso, Female, 36 years)*

*“Last month, my time was due for check-up but I couldn’t go because there was no one available to escort me and when I told the doctor about my situation, he then suggested to shift my time for check-up to 6 months instead of 4 months and I told him that is not the best way because that will pose a lot of challenge for me”. (Visually Impaired, Kumasi, Female, 58 years)*

###### Stigma and discrimination towards persons with disabilities

Stigma and discrimination was a significant factor both from the community and even within family members. This discrimination took many forms, manifested as negative attitudes stemming from a lack of knowledge about disability and prejudice surrounding the SRH abilities of PwDs.

“*The stigma is a very huge challenge so they will be like how does sex concern someone who can’t see? So, all this stigma is huge and it makes it very difficult for PwDs to access SRH information and services in general. That is the basic challenge and all other challenges fall under this”. (Visually Impaired, Kumasi, Male, 31 years)*

“*I can also say that sometimes, it is about discrimination. Even though there is publicity about persons with disabilities. For some people with disabilities, when they get pregnant, they think they will be stigmatised for being pregnant. Some people can even say that ‘eii … do you also have sex!’ and other comments. Such people think if you are disabled, you are denied those rights but that’s not the case. All these prevent PwDs from accessing sexual and reproductive services because of the unnecessary questions posed to them sometimes”. (Visually Impaired, Kumasi, Female, 21 years)*

##### Health facility–systemic barriers

The health facility-level barriers shared by PwDs as factors inhibiting their use of SRH services were physical barriers at the health facility (such as unfriendly health infrastructure), lengthy waiting periods, health providers’ negative attitudes towards the SRH rights and abilities of PwDs, lack of privacy, and health policy-related barriers.

###### Disability-unfriendly health facility infrastructure

At the facility level, several participants expressed the challenges they encountered in navigating the premises due to the unfriendly nature of the building.

*“…Also, they have to work on the hospital buildings. Currently, at the hospital that I attend, I have to be lifted if I want to get inside. So, they should consider working on these infrastructural challenges”. (Visually Impaired, Offinso, Female, 58 years)*

*“Sometimes, it is difficult for us to move around and physically access health facilities to get access to SRH information and services”. (Physically Disabled, Kumasi, Male, 56 years)*

###### Long waiting times at health facilities

Some participants also mentioned the exhaustion and difficulty they experienced while receiving SRH services due to long waiting periods in health facilities. Specifically, a few participants reported waiting for more than 8 h to see doctors, which ultimately demotivated them from seeking subsequent care even if the need arose.

*“The only challenge was that there is a lot of delays on arrival at the hospital. I go there at 6am, and sometimes come home at 5pm”. (Visually Impaired, Kumasi, Male, 45 years)*

*“For visually impaired people, most of the time, we plead with people to escort us to the hospital so when we come, they should consider and take care of us on time so that we won’t waste the time of those who normally escort us to the hospital”. (Visually Impaired, Kumasi, Female, 58 years)*

###### Health providers’ negative attitudes towards sexual and reproductive rights and abilities of PwDs

Another health facility-related barrier that PwDs discussed was the negative attitude of some HPs. Some PwDs expressed the challenge they faced in visiting hospitals to seek SRH services due to the unfavorable sentiments or treatments they received from HPs when seeking treatments for STIs.

“*Currently, our biggest challenge has to do with the disrespect that some HPs show to us. When you go to the hospital as a PwD with STIs, the doctors pass silly comments like ‘you too?’ Being disabled doesn’t mean that your penis or vagina doesn’t work. A friend of mine experienced something similar. He is physically disabled and had an STI. The doctor who attended to him passed the comment that, ‘You don’t have strength and you are having sex’. Another lady also faced such disrespect from a nurse who said, ‘you these disabled people … you are so promiscuous. How can you be pregnant?’ … My friend got angry and asked that is it bad for her to give birth as a PwD? Things like these discourage PwDs from seeking SRH healthcare”. (Visually Impaired, Offinso, Male, 55 years)*

###### Lack of privacy

Privacy for PwDs was identified as another health facility-level barrier that emerged from the data. Several participants indicated that, in general, once an individual becomes disabled, their privacy is also affected. When it comes to utilizing SRH services, certain matters are sensitive, prompting PwDs to consistently seek confidential and private consultations with HPs. Additionally, some PwDs shared how privacy concerns can vary depending on the type of health facility they are accessing.

“*When it is time for consultation, privacy must be adhered to. So, my personal assistant or whoever am walking with must leave us for you to talk to me. But then, they [health professionals] always want to involve our caregivers, meaning they don’t respect our privacy”. (Visually Impaired, Kumasi, Male, 31 years)*

*“…At the government hospitals, there is no privacy in the consulting rooms because more than one person might be there. But when it comes to the private herbal facilities, it is only one person in the consulting room. And so, for sure, I will be able to easily communicate my issues to those at the private facility than at the government hospital because at the hospital, I will feel shy to talk about my reproductive health issues. For instance, I went to [Name of Herbal Clinic] when I had an STI. Over there, it was only one doctor who was in the consulting room with me. I was able to tell him everything and all of the symptoms”. (Physically Disabled, Kumasi, Male, 36 years)*

###### Health policy barriers

The NHIS was introduced in Ghana with the aim of eliminating out-of-pocket payments and providing protection to vulnerable populations, including PwDs. However, some participants shared their experiences highlighting the ineffectiveness of the NHIS, which is leading to their inability to afford out-of-pocket costs for SRH services.

*“Another challenge is that the NHIS as I said earlier is not free so it makes it difficult for PwDs to have access to SRH services. I am a credit vendor and I don’t earn much so if I go to the hospital and they collect 30 Ghana cedis from me, I can’t go to the hospital again because I don’t earn that amount of money every day. I earn less than 30 cedis a day. I remember when I went to the hospital some time ago, the NHIS didn’t cover my bills. The only thing the NHIS covered was the hospital card they gave to me. They collected money when I went to the laboratory for laboratory test”. (Physically Disabled, Kumasi, Female, 27 years)*

*“Since the health insurance came, I have registered for it about six times. It has been a while that I renewed it because when you take it to the hospital, you still pay for most of the medications. I don’t understand why some of the medications are not covered by the health insurance. Now, I have come to the realization that God is my only health insurance”. (Physically Disabled, Offinso, Male, 46 years)*

#### Enablers to the use of sexual and reproductive health services among persons with disabilities

##### Individual level factors

Two main individual-level factors, namely self-efficacy and knowledge about the importance of SRH services, emerged as key motivators for some PwDs to seek SRH services.

*“… For me, when I got pregnant, I went to the hospital so that I could deliver there. I knew that by going to the hospital, the HPs could easily detect if there is something wrong with my child. I started going to the hospital when I was three months into the pregnancy. I was able to attend all of the ANC sessions after my third month of pregnancy. The reason was that, I wanted to ensure the health of both my child and myself”. (Visually Impaired, Offinso, Female, 33 years)*

*“Sometimes, because I can freely express myself, I am able to tell the doctors or health providers exactly what I want but others are not like me. I cannot speak for others but for me, I can easily access SRH services and information”. (Visually Impaired, Kumasi, Male, 38 years)*

##### Family/community level factors

The family/community level factor that assisted some PwDs to seek SRH was support from family members. While some of the PwDs recounted how they lack family or social support to seek SRH, some of the participants shared the opposite. Particularly, some indicated how their significant others such as partners were supportive, helping them to seek SRH services.

*“In my case, I attended all my ANC sessions because my partner was supportive. As a result of that, the health providers were able to check the health status of my child. Because of that, I had smooth childbirth for all my pregnancies. The moment I get there [health facility], the child comes out smoothly”. (Physically Disabled, Offinso, Female, 46 years)*

“*Going to the hospital during that time, it was my first born who used to assist me to attend my ANC appointments”. (Visually Impaired, Kumasi, Female, 27 years)*

##### Health facility level factors

Four key health facility level factors were also discussed by the participants as enablers to their use of SRH services. These were: (a) appropriate means of communicating SRH information to PwDs, (b) disability-friendly infrastructure, (c) health professionals’ positive attitude and preferential treatment towards PwDs, and (d) NHIS subscription.

###### Appropriate communication

Some of the participants shared how the use of local languages (e.g., Twi and Hausa) instead of English made it easier for them to understand SRH information provided by HPs.

*“I don’t think language was a challenge because they deliver SRH education in the local language (Twi). So, it is easy for us to understand the information that they provide to us and therefore put that into practice”. (Physically Disabled, Offinso, Female, 36 years)*

*“I think that the SRH information that we receive from the radio and information centre from the HPs is easy to understand. They usually speak Twi or Hausa which are the dominant languages spoken in this community”. (Visually Impaired, Kumasi, Male, 45 years)*

###### Disability-friendly infrastructure

Some of the participants expressed that certain health facilities, especially the teaching hospital they visited, were disability-friendly, making it easier for them to navigate.

*“With respect to the facility, the building is friendly for PwDs because they have a place where those in wheel chairs can pass to access the health facility”.* (*Physically Disabled, Kumasi, Female, 27 years)*

“*As for [Name of health facility] it is user-friendly; it is their washroom which is not tidy”. (Visually Impaired, Kumasi, Female, 58 years)*

*“The health facility here has a disability-friendly infrastructure. They don’t only have steps [staircase]. There are ramps and slopes that makes it easy for me to access it”. (Physically Disabled, Offinso, Male, 46 years)*

###### HPs positive attitude and preferential treatment

While some participants described experiencing negative attitudes and treatment from HPs, others indicated that some HPs demonstrated respect, compassion, and preferential treatment towards them. Several participants specifically described the professional traits exhibited by these HPs.

“*The health professionals are really ‘professionals’. They care for us with utmost respect”.* (Physically Disabled, Offinso, Male, 50 years)

*“… However, there are others HPs too that are PwD-friendly. For instance, about two weeks ago, I was feeling ill so I took my national health insurance card and went to the clinic. I went along with my last child. So, when the nurse saw me with the child, she brought me a chair and then went in to get me my folder. After that, she led me to the doctor after they had checked my blood pressure and other vitals. She skipped the queue for me because of my condition”. (Visually Impaired, Offinso, Male, 55 years)*

*“They were very kind to me. Most times, when I go to the health facility, the HPs prioritize me. So, they will allow me to jump the long queue to prevent delays at the facility. I think it is the government that has given them that directive to care for us first before any other person in a queue at the health facility”. (Physically Disabled, Offinso, Male, 57 years)*

###### Health policy: National Health Insurance Scheme subscription

Amidst the critiques expressed by most PwDs regarding the effectiveness of the NHIS, some participants indicated that it has been supportive in covering a portion of the costs associated with SRH care.

*“The health insurance is good because it makes access to sexual and reproductive healthcare extremely easy. But I was not utilising it because I don’t get sick often. The only time I used it was when I had gonorrhoea”. (Physically Disabled, Offinso, Male, 35 years)*

*“Had it not been for the health insurance, a lot of PwDs would not be able to afford the cost of seeking SRH services. So, if they have the insurance, they can go there and if there is the need to pay, they would just pay something minimal”. (Visually Impaired, Offinso, Male, 55 years)*

#### Triangulation of findings

The results from both phases of the study were triangulated as shown in Table 3.

**Table 3:** Integration of qualitative and quantitative findings.

Theme Barriers	Sub-theme	Quantitative findings	Illustrative qualitative quote	Synthesis
Individual level	Financial challenges	45.5% of the respondents indicated that high cost of SRH is a barrier.	“*These days, things are difficult in terms of economic issues (financial constraints). It is not all the time that your partner can get money to support you to access SRH services. You know that most of these things you need to pay before you can access them. So if there is no money what can you do. You only sit down and pray for the Good Lord to heal you.” (Physically Disabled, Kumasi, Female, 33 years)*	The findings underscore the need to empower PwDs economically. Additionally, it is crucial to revitalize the National Health Insurance Scheme to facilitate PwDs’ access to SRH services.
Family/community level	Stigma and discrimination	27.3% indicated that stigma and discrimination is a barrier to their use of SRH services.	*“You will be there and people will be passing derogatory statements like how can a PwD get pregnant. So, it is not the best.” (Visually Impaired, Offinso, Female, 51 years)*	It is crucial to employ a variety of campaign techniques to enhance community and family members’ knowledge of disability issues and the rights of PwDs. One approach is to broadcast a few common stories of PwDs to the general public through suitable channels, or publish them on social media, focusing on the capabilities and sexual rights of PwDs.
Health facility level	Physical barriers	54.6% indicated that disability-unfriendly infrastructure was a barrier to their successful use of SRH services.	*“…But there was a point that I had to be carried because the stairs were too high. So, I think that the health facility must think of measures to make the built environment much friendly for PwDs to easily access it”*. *(Physically Disabled, Offinso, Male, 57 years)*	To meet the needs of PwDs, certain health facilities must be renovated to ensure disability-friendly environments. Additionally, accessible design features should be incorporated into the design of new health facilities.
	Long waiting times at health facilities	45.5% agreed that long wait times are a barrier to their use of SRH services.	*“Sometimes, at the hospital, if you are not the enlightened type, you will be there and they will serve other people at your expense… leaving you in the queue”. (Visually Impaired, Offinso, Female, 51 years)*	As enshrined in the Disability Act 715 of Ghana, the SRH of PwDs should be prioritized, enabling them to access SRH services.
	HPs negative attitude towards PwDs	27.3% indicated that discrimination by health professionals is a barrier.	*“…And we the blind they do not want to make time for us, especially when prescribing drugs for us. They do not regard us and want to talk to people who brought us. This affects us. The way they communicate with us is not good. I had a personal experience with the attitude of healthcare providers. The nurse talked to me anyhow and I even reported him to the doctor and the doctor warned him because he was far younger than me*”. *(Visually Impaired, Kumasi, Male, 45 years)* *“I remember there was a time I went to the hospital and the doctor was like take this prescription. I heard you have seven children. You do not have money and you have given birth to seven children. I did not reply him because I felt he was not being reasonable. Like I talked about the doctor who said, I do not have money but I have given birth to many children, it is a clear indication of discrimination. So, health providers must desist from such behaviours. They must treat us with utmost respect.” (Physically Disabled, Kumasi, Male, 47 years)*	It is important to intensify training for HPs to enhance their understanding of disability. Additionally, efforts should be made to develop appropriate communication skills in order to provide high-quality care to PwDs.
	Lack of privacy	18.2% indicated that lack of privacy was a barrier to their use of SRH services.	*“When it is time for consultation, privacy must be adhered to. So, my personal assistant or whoever I am walking with must leave us for you [health professional] to talk to me. But then, they[health professionals] always want to involve them, meaning they do not respect our privacy”*. (*Visually Impaired, Kumasi, Male, 31 years)*	Due to the sensitive nature of SRH issues, the lack of privacy could hinder PwDs from sharing their problems, potentially affecting their treatment and future utilization of services. It is imperative for the government and health facility managers to take pragmatic steps to ensure that the privacy and rights of PwDs are safeguarded during consultations.
Enablers				
Individual level	Self-efficacy and knowledge on the importance of SRH services		*“…And so, when I went to the hospital and it was confirmed that I was pregnant, I started ANC attendance. I believe that was one of the things that made my pregnancy easier to bear. For the first one month, I was not aware of the pregnancy until I went to the hospital”. (Visually Impaired, Offinso North, Female,* 33 years)	The self-efficacy of PwDs should be improved to facilitate easier access to SRH services. It is crucial to provide PwDs with additional SRH informational resources. To educate PwDs about the significance of SRH, the government should support programs specifically tailored to this population.
Family/community level	Support from family members	41.2% agreed that support from care givers enabled them to seek SRH services.	*“…. So, it was normal and by God’s grace, I had someone I was going to antenatal bookings with. So, the support I had made my pregnancy days okay and a smooth one for me”. (Visually Impaired, Kumasi, Female, 21 years)*	Family members and other significant others should be encouraged to support the daily lives of PwDs, including their access to SRH services. However, it is crucial to educate them about the SRH rights of PwDs. This will enable them to ensure that PwDs are provided with the highest level of privacy during consultations.
Health facility level	HPs positive attitude and preferential treatment	66.2% agreed that healthcare providers’ positive attitudes enabled them to seek SRH services 40.4% indicated that the preferential treatment they received at the health facilities enabled them to seek SRH services.	“*To be honest, the health professionals at [Facility Name]were so kind to me when I had my condition. They were so encouraging and supportive. They treated me with a lot of dignity and respect. I think that if all the hospitals were like that, then it will be good”. (Physically Disabled, Kumasi, Male, 45 years)* *“In fact, when I go to the facility, they even allow me to be cared for first before ‘abled’ people. Some PwDs feel shy to overtake abled people when it comes to seeking healthcare.” (Physically Disabled, Offinso, Male, 46 years)*	The positive attitude exhibited by some of the healthcare providers presents an opportunity for behavioral change and professionalism towards service provisions to PwDs.
	National health insurance	64.7% agreed that the NHIS helped them to seek SRH services.	*“The health insurance has been effective and useful in helping us prevent and seek early treatment for any sexually transmitted infections. Because you have the insurance, you can easily go to the health facility, and receive comprehensive care”*. *(Physically Disabled, Offinso, Male, 57 years)*	It is important to strengthen the NHIS to ensure that it fulfills its intended purpose of improving healthcare access for the vulnerable in society.

## Discussion

This mixed-methods study examined the factors influencing the utilisation of SRH services by PwDs, as well as the enablers and barriers they face in accessing these services. The quantitative findings revealed a low usage (33.8%) of SRH services among PwDs. A multivariate analysis showed that sex, marital status and travel time to health facility were significant predictors of SRH services usage. The qualitative findings identified individual, family/community, and health facility level factors as enablers and barriers to SRH service utilisation. The main enablers included positive attitudes of some HPs, NHIS subscription, support from caregivers, and preferential treatment at the hospital. The main barriers consisted of unfriendly disability infrastructure, long waiting time, high SRH cost, and discrimination by some HPs. These findings are consistent with previous studies in other LMICs such as Nepal (44), Cameroon (45), and Ethiopia (46) which also reported low utilisation of SRH services by PwDs and similar factors influencing their access. The conceptual framework (Figure 1) guiding this study suggests that client characteristics affect their use of SRH services, which in turn have an impact on their SRH outcomes (25, 26, 28). Specifically, males were less likely to use SRH services than females, possibly due to the perception that SRH is a ‘female affair’. Moreover, married PwDs were more likely to use SRH services than those who were never married. Marital status has been found to significantly influence the utilization of maternal and child health services among PwDs (44). Married individuals are more likely to give birth and, consequently, are more inclined to seek and utilize maternal and child health services. Moreover, it is possible that some married PwDs are utilizing family planning methods to achieve their desired fertility outcomes. These findings suggest a heightened demand for family planning and reproductive healthcare services among married couples with disabilities, highlighting the importance of addressing their specific needs and ensuring accessible and inclusive healthcare provision.

From the qualitative findings, one of the main barriers to the use of SRH services was the cost, as also found in previous studies in Malawi and Ghana (11, 23, 24). Disability and poverty are intricately intertwined, as disability can be “both a cause and a consequence of poverty,” exacerbating vulnerability and exclusion (47). PwDs typically have lower levels of education, career opportunities, and income than the general population, limiting their abilities to afford the cost associated with SRH services (44, 45, 48). Another barrier to the use of SRH services was the lack of knowledge or misinformation about some SRH products and their side effects. Some of the participants reported that they did not use SRH services because of their previous experience or their partners’ opinions on the negative effects of some SRH products. Others also attributed their non-use to religious beliefs and misconceptions surrounding SRH services. To address this, SRH education should be intensified to tackle myths and misconceptions surrounding SRH product use, as some of the participants indicated their knowledge on the importance of SRH services was a facilitator to their use. As Ganle et al. (19) argued, PwDs need to be better informed on their sexual rights and the availability of treatments for SRH. This is crucial to combat PwDs’ lack of self-efficacy and curtail misconception of some SRH services.

The participants reported several challenges at the family/community level that hindered their access to SRH services. These included transportation issues, inadequate social support, and stigma and discrimination. These challenges have been well documented in previous studies in Uganda and Ghana (17, 24). The participants indicated that they often struggled to find suitable means of transport to health facilities to seek SRH services. Moreover, they lacked adequate social support from their families and caregivers, which has also been reported in previous studies in Malawi and Senegal (11, 14). However, some of the participants in this study stated that they were able to access SRH services due to the support networks they had. Therefore, it is important to ensure that PwDs have ample support systems to facilitate their access to SRH care, as implemented in some high income countries like Australia (49).

In line with previous evidence in Ghana, Uganda and elsewhere (17, 19, 20) another challenge that the participants faced was the stigma and discrimination from some community members regarding their use of SRH services. PwDs are often subjected to differential treatment regarding their SRH due to social beliefs that they should not engage in or are incapable of engaging in sexual activity. Consequently, society perceives PwDs as not needing SRH services, leading to frequent instances of humiliation and abuse in public settings. These societal perspectives greatly contribute to the marginalization of PwDs (19). Therefore, it is crucial to intensify education on the sexual rights and abilities of PwDs to challenge these misconceptions and promote inclusivity and respect.

The participants also encountered barriers at the health facility level. These included physical barriers to facilities, long waiting time, high cost of SRH services, and discrimination by some HPs. These barriers have also been found in previous studies in Ghana and United States of America (24, 50). Although the Disability Act 715 of Ghana recommends the need to make public buildings including health facilities disability-friendly, this is not always the case in most health facilities. Some participants indicated that the higher-level health facilities such as the teaching hospital were disability-friendly, but most of the participants also revealed that the lower level facilities were not disability-friendly. This reiterates the need to make the health facilities more disability-friendly (19). Long waiting times in public hospitals are not peculiar to only PwDs but among all healthcare users. This is partly due to the doctor or healthcare provider-to-patient ratio. For example, in Ghana, the World Bank data shows that currently there are 0.2 physicians per 1,000 population, and 3.6 Nurses and Midwives per 1,000 people (51). Due to the disability-unfriendly nature of certain health facilities, long waiting times for PwDs pose a significant problem. These extended waiting periods can cause discomfort, particularly when combined with the lack of disability-friendly washrooms in these facilities. Consequently, this can deter PwDs from further utilizing these facilities, even when the need arises (17, 52, 53).

A number of the participants indicated that they received preferential treatment from some HPs due to their disability status. This was another factor that facilitated their access to SRH services. However, preferential treatment of PwDs has been interpreted differently in the literature. Some PwDs perceive this as a sign of pity from HPs, while others see this as an opportunity to seek prompt care (15). It is important to standardize the prioritization of PwDs throughout the country to make all patients aware and avoid the feeling that PwDs are over prioritized. As Soule and Sonko (53) suggested, units within health facilities could be specifically dedicated to treating PwDs to ensure that the services there are adapted to their special needs, however, this should be done strategically to avoid further discrimination.

The attitude of HPs towards PwDs varied from both positive and negative. Some PwDs stated that HPs’ negative attitude served as a barrier to their use of SRH services, while others revealed how the positive attitude and appropriate communication were enabling factors to their use of SRH services. Previous evidence suggest that HPs with more experience in the provision of healthcare to PwDs had more positive attitudes towards them (54, 55). This finding suggests that there is an opportunity to increase HPs’ positive attitudes toward PwDs through training on disability-related issues and care. The participants also reported that lack of privacy and confidentiality was a major barrier to the use of SRH services, as found by Burke et al. (14) in Senegal. Since SRH issues are very sensitive, some PwDs might feel uncomfortable to access SRH or feel shy to share their medical history for fear of being judged. The government and health facility managers should take pragmatic steps to ensure that the privacy and rights of PwDs are assured during care.

The impact of the NHIS scheme on the accessibility of SRH care for PwDs was also discussed by the participants. Within the health outcomes model guiding this study, the nature and effectiveness of health interventions have a great influence on the SRH seeking behaviour and outcomes of PwDs. However, the majority of the participants indicated that the NHIS is ineffective, while a few others reported that it had helped them access SRH services. Previous studies have reported the impact of NHIS on SRH use among young PwDs in Ghana (22). This calls for a review of the NHIS to make it more effective to meet the need of PwDs.

## Implications for policy and practice

The findings of this mixed-methods study on the enablers and barriers to the use of SRH services by PwDs have various implications for policy and practice. First, the results highlight the need to empower PwDs economically. Second, they suggest the need to revitalize the NHIS to make it more effective in aiding PwDs’ access to SRH services. Third, they indicate the importance of improving PwDs self-efficacy and providing more SRH informational resources to them. The government should support programs that are specifically geared at educating PwDs about the significance of SRH. Fourth, they underscore the role of family members and other significant others in supporting the daily living of PwDs including their access to SRH services. Community sensitization campaigns should also be employed to raise community and family members’ knowledge on PwDs rights. Moreover, to meet the needs of PwDs, some of the health facilities should be renovated to make them more disability-friendly. Furthermore, more training opportunities for HPs are highly recommended to increase their understanding of disabilities and appropriate communication skills towards PwDs.

## Strength and limitations

The strengths and limitations of this study should be discussed. First, the use of a mixed-methods approach enabled the researchers to gain a deeper understanding of the experiences of PwDs on their access to SRH services (56). The researchers also collaborated with the disability organizations in the Kumasi Metropolis and Offinso North district to foster trust and relationships between the researchers and PwDs. The quantitative phase of the study had a relatively large sample size, which makes generalizations of the study findings to PwDs in the Ashanti Region possible. However, the possibility of social desirability biases cannot be ruled out since some of the issues were self-reported. Also, there was no single word for SRH in Twi language. To overcome this, the RAs and the principal researcher took their time to clarify what SRH meant to the participants. Finally, only two groups–persons with physical disabilities and those with visual impairments were considered in this study.

## Conclusion

This study has demonstrated that PwDs in Ghana have relatively low utilisation of SRH services. The use of SRH services among PwDs is influenced by various factors at the individual, family, community and health facility levels, which can act as both enablers and barriers. To improve the accessibility and quality of SRH services for PwDs, several recommendations can be made. First, stigma and discrimination towards PwDs should be reduced through community education and sensitization on the SRH rights and abilities of PwDs. Second, health facilities and buildings should comply with the Disability Act 715 to make public spaces more disability-friendly. Third, HPs should receive more training opportunities on disability-related issues to ensure adequate high quality care and support. Fourth, the NHIS should be strengthened to make it more effective in increasing healthcare affordability. These recommendations could enhance the utilisation of SRH services among PwDs and contribute to their well-being and empowerment. Further research could explore SRH outcomes among PwDs and strategies to improve these outcomes in Ghana.

## Data availability statement

The raw data supporting the conclusions of this article will be made available by the authors, without undue reservation.

## Ethics statement

This study followed the ethical guidelines and approval from three institutional review committees: the Ghana Health Service (GHS) Ethics Review Committee (GHS-ERC: 005–0621), the Komfo Anokye Teaching Hospital (KATH) (KATH-IRB/RR/101/21), and the James Cook University (JCU) Human Ethics Committee (H8531). Additionally, the Regional Health Directorate in Kumasi and the Offinso North District Health Directorate in Akumadan also endorsed the ethics approval forms. The leaders of two disability groups in Kumasi Metropolis and Offinso North District also consented to the study. Furthermore, participants’ anonymity and confidentiality were ensured. Specifically, before the administration of the study instrument, a document stating the study objectives, anonymity, confidentiality and merits of the study were explained to respondents. The respondents were made aware that the information provided is purely for academic work and that their identities will not be revealed to the general public. Respondents were also informed about their right to opt out of the study at any given time if they so desire. Written and verbal informed consent were sought from all the respondents. The patients/participants provided their written informed consent to participate in this study. Written informed consent was obtained from the individual(s) for the publication of any potentially identifiable images or data included in this article.

## Author contributions

A-AS, BM-A, KM-R, AM-A, and TE: conceptualization and writing—review and editing. A-AS: writing—original draft preparation. BM-A, KM-R, AM-A, and TE: supervision. A-AS and TE: funding acquisition. All authors approved it for publication. All authors contributed to the article and approved the submitted version.

## Conflict of interest

The authors declare that the research was conducted in the absence of any commercial or financial relationships that could be construed as a potential conflict of interest.

## Publisher’s note

All claims expressed in this article are solely those of the authors and do not necessarily represent those of their affiliated organizations, or those of the publisher, the editors and the reviewers. Any product that may be evaluated in this article, or claim that may be made by its manufacturer, is not guaranteed or endorsed by the publisher.

## Supplementary material

The Supplementary material for this article can be found online at:

https://www.frontiersin.org/articles/10.3389/fpubh.2023.1232046/full#supplementary-material

## Abbreviations

aOR: adjusted odds Ratio
CI: Confidence Interval
COREQ: Consolidated criteria for reporting qualitative research
GHS: Ghana Health Service
GSS: Ghana Statistical Service
JCU: James Cook University
KATH: Komfo Anokye Teaching Hospital
LMICs: Low- and middle-income countries
NHIS: National Health Insurance Scheme
PwDs: Persons with disabilities
RAs: Research assistants
SRH: Sexual and reproductive health
UN: United Nations
HPs: Healthcare providers
WHO: World Health Organization
